# Supplementary material for: Advancement in long-distance bird migration through individual plasticity in departure
Source: Nat Commun. 2021 Aug 6;12:4780. doi: 10.1038/s41467-021-25022-7 (PMC8346503; doi:10.1038/s41467-021-25022-7)
Supplement: Supplementary file 1 — Supplementary Information [file 41467_2021_25022_MOESM1_ESM.pdf]

Supplementary Information for

Advancement in long-distance bird migration through individual plasticity in departure

Jesse R. Conklin, Simeon Lisovski and Phil F. Battley

Correspondence to: conklin.jesse@gmail.com or simeon.lisovski@awi.de or P.Battley@massey.ac.nz

This PDF includes:
Supplementary Figures 1–3
Supplementary Tables 1–3
Supplementary Methods: Environmental data analyses

Supplementary Figure 1. Overall, individual bar-tailed godwits showed a negative trend of –0.46 d/yr in departure date from New Zealand (95% CrI: –7.47 to +6.09). For 69% of individuals (86 of 124 with ≥3 years data), the probability of a negative slope was higher than a non-negative slope (50% cutoff indicated by dashed line).

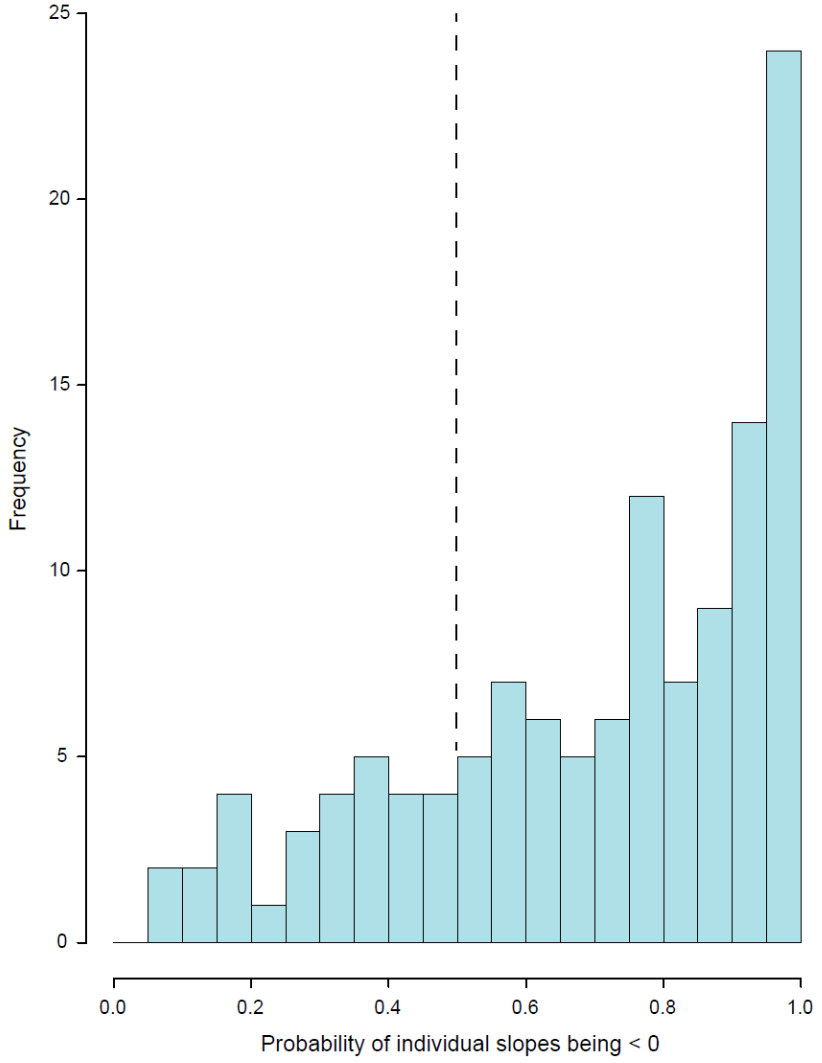

Supplementary Figure 2. Size distribution (length of exposed culmen, mm) of marked bar-tailed godwit individuals departing the New Zealand study site on migration each year 2008–2020. Darker dots indicate annual means by sex (female = purple, male = yellow). Numbers indicate sample sizes by sex and year. There was no trend in bill length across years (linear regression; females: slope = 0.12, t = 1.25, p = 0.21; males: slope = –0.11, t = –1.56, p = 0.12).

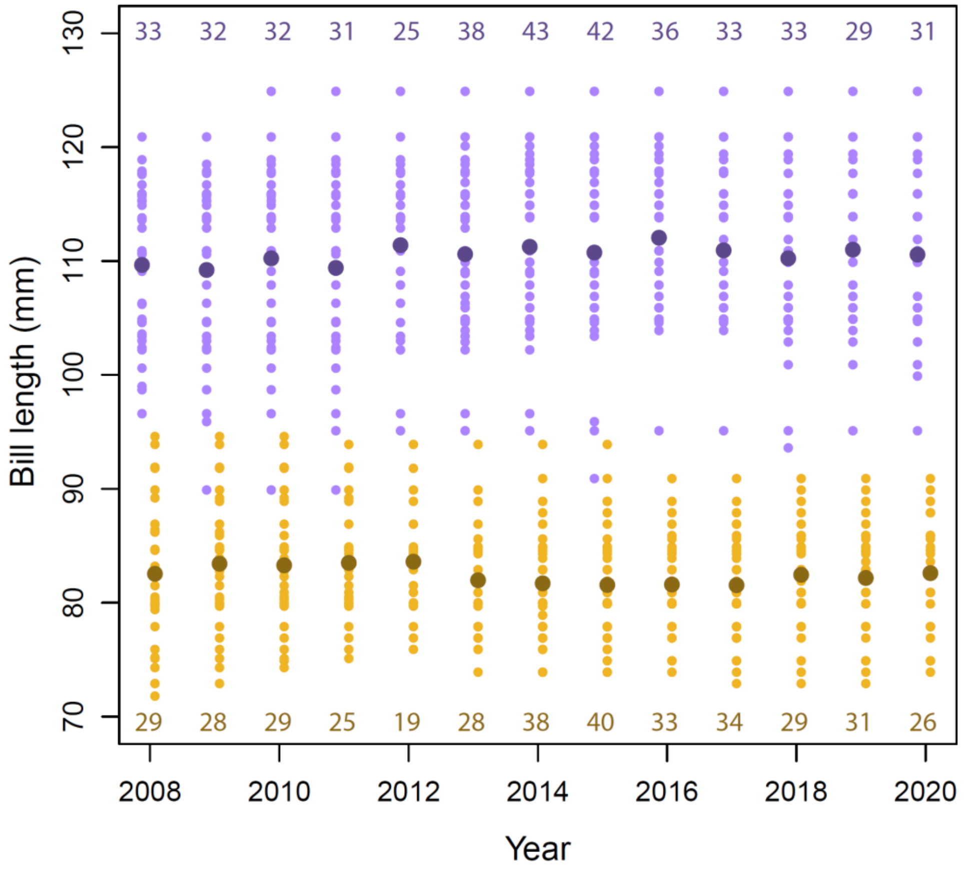

Supplementary Figure 3. Estimated breeding locations based on geolocator-tracking. **a** Breeding locations for 16 bar-tailed godwits tracked from New Zealand to Alaska with BAS MK14 geolocators in 2008 and 2009. For 13 individuals breeding below the Arctic Circle, estimated location (orange dots) plus 95% range of latitude and longitude (vertical and horizontal black lines, respectively) is shown. For three individuals, the bird's last discernible location before traveling north to 24-hour daylight is shown (grey dots plus 95% range). These three birds are presumed to have bred on the North Slope of Alaska (green dots), but location estimates are not possible. **b** Breeding locations for godwits tracked to Alaska with Intigeo-C65K (blue dots; *n* = 17) and Biotrack (red dots; *n* = 4) geolocators in 2013 and 2014. Dots indicate highest-likelihood locations and black lines indicate the range of latitude and longitude with 95% probability to contain the true location. Yellow polygon shows the known breeding range. Some points with very similar estimates are shifted slightly for clarity. While estimates are increasingly imprecise at higher latitudes, individuals can be assigned to northern or southern latitude groups (>/<64°N; dashed line) with high confidence regardless of method **(a, b)**. Maps made with Natural Earth.

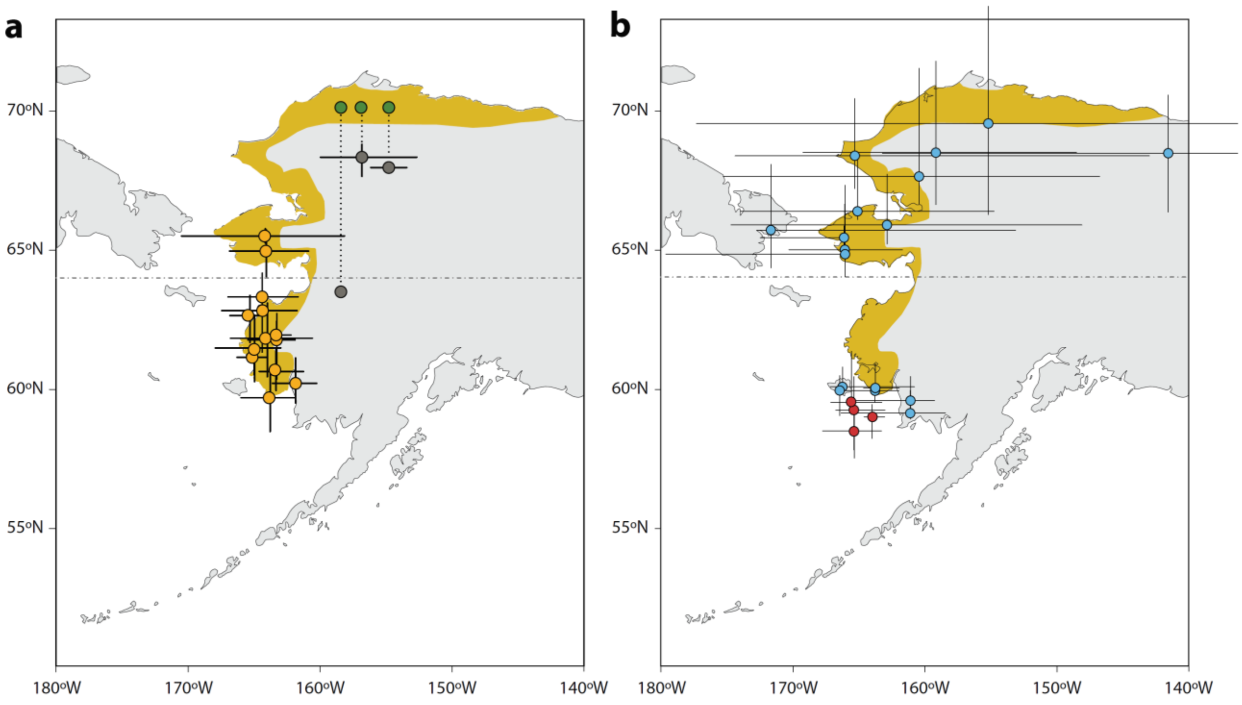

Supplementary Table 1. Results from within-subject centering analysis of bar-tailed godwit departure from New Zealand across the years 2008–2020 (see Fig. 2b and 3b). For equation 2 (eq2; van de Pol and Wright 2009), significant result (in bold) indicates that the estimated slope (d/yr) differs from zero ($p < 0.05$) in a linear mixed model. For equation 3 (eq3; van de Pol and Wright 2009), a non-significant result indicates that between- and within-subject slopes are not distinguishable (i.e. that within-individual changes can fully explain population-level advancement). **a** includes all marked individuals with ≥3 years of departure data. **b** includes the subset of 34 individuals with known Alaska breeding locations from geolocator tracking (see Fig. 3a) and ≥3 years of directly-observed departure data.

a Full dataset $n = 124$

				**eq2**			**eq3**
**Parameter**	**Estimate**	**SE**	***t***	***p***	**2.5%CI**	**97.5%CI**	***p***
Intercept	76.828	0.228	336.94	< 0.0001	76.380	77.276	
Between-subject	**–0.463**	0.082	–5.62	**< 0.0001**	–0.625	–0.302	0.784
Within-subject	**–0.428**	0.100	–4.29	**< 0.0001**	–0.624	–0.232	

b Individuals with known breeding locations

North (>64°N) $n = 15$

				**eq2**			**eq3**
**Parameter**	**Estimate**	**SE**	***t***	***p***	**2.5%CI**	**97.5%CI**	***p***
Intercept	81.052	0.474	170.85	< 0.0001	80.110	81.994	
Between-subject	**–0.827**	0.186	–4.44	**< 0.0001**	–1.197	–0.458	0.241
Within-subject	**–0.500**	0.206	–2.44	**0.017**	–0.908	–0.093	

South (<64°N) $n = 19$

				**eq2**			**eq3**
**Parameter**	**Estimate**	**SE**	***t***	***p***	**2.5%CI**	**97.5%CI**	***p***
Intercept	74.304	0.515	144.28	< 0.0001	73.283	75.325	
Between-subject	–0.192	0.199	–0.97	0.335	–0.586	0.202	0.255
Within-subject	**–0.522**	0.209	–2.50	**0.014**	–0.937	–0.108	

Supplementary Table 2. Change in timing (d/yr) of **(a)** snowmelt and **(b)** Normalized Difference Vegetation Index (NDVI) in the Alaska breeding range of bar-tailed godwits (North >64°N, South <64°N; see Fig. 3) during the entire study period (2008–2020) and the shorter period of geolocator-tracking (2008–2014).

a Snowmelt

**Period**	**Region**	**Median**	**SE**	**2.5%CI**	**97.5%CI**
2008–2020	All Alaska	–0.651	0.0001	–0.663	–0.639
	North	–0.235	0.0002	–0.248	–0.222
	South	–1.926	0.0003	–1.951	–1.900
2008–2014	All Alaska	–0.174	0.0003	–0.200	–0.147
	North	0.394	0.0003	0.367	0.421
	South	–1.913	0.0007	–1.976	–1.849

b NDVI

**Period**	**Region**	**Median**	**SE**	**2.5%CI**	**97.5%CI**
2008–2020	All Alaska	–0.944	0.0001	–0.955	–0.933
	North	–0.467	0.0001	–0.479	–0.456
	South	–2.296	0.0003	–2.320	–2.273
2008–2014	All Alaska	–0.088	0.0003	–0.627	–0.558
	North	0.579	0.0003	0.555	0.603
	South	–1.955	0.0006	–2.012	–1.897

Supplementary Table 3. Change in phenology of all stages of northward migration and breeding of geolocator-tracked bar-tailed godwits during 2008–2014, derived from linear mixed models including individual as a random effect and breeding region (North: >64°N, South: <64°N; see Fig. 2a) as a fixed factor. Slopes significantly different from zero ($p < 0.05$) are in bold. Sample size is 50 total tracks of 36 individuals (except for Start incubation = 41/29); number of tracks per year is shown in Fig. 4. Note that northern breeders were *ca.* 7 days later than southern breeders on the initial flight from New Zealand, but *ca.* 12 days later for the second flight to Alaska, after a longer stay in the Yellow Sea.

**Migration stage**	**Fixed effect**	**Estimate (d)**	**SE**	***t***	**d.f.**	***p***
Depart New Zealand	Intercept	1781.881	572.892	3.11	46.4	0.003
	Year	**–0.845**	0.285	–2.97	46.4	**0.005**
	Breed South	–7.456	1.605	–4.65	33.8	< 0.0001
Arrive Yellow Sea	Intercept	1870.705	537.224	3.48	47.0	0.001
	Year	**–0.885**	0.267	–3.31	47.0	**0.002**
	Breed South	–6.879	1.552	–4.43	34.2	< 0.0001
Depart Yellow Sea	Intercept	805.954	517.473	1.56	42.9	0.127
	Year	–0.333	0.257	–1.30	42.9	0.202
	Breed South	–12.156	1.612	–7.54	28.6	< 0.0001
Arrive Alaska	Intercept	553.210	469.613	1.18	41.9	0.245
	Year	–0.205	0.233	–0.88	41.9	0.384
	Breed South	–11.664	1.492	–7.82	30.6	< 0.0001
Start incubation	Intercept	25.926	809.541	0.03	38.0	0.975
	Year	0.066	0.402	0.16	38.0	0.870
	Breed South	–10.129	2.092	–4.84	38.0	< 0.0001
Duration Yellow Sea	Intercept	–1348.297	607.923	–2.22	46.8	0.031
	Year	**0.693**	0.302	2.29	46.8	**0.026**
	Breed South	–5.006	1.734	–2.89	30.4	0.007

Supplementary Methods: Environmental data analyses

Timing of breeding in Arctic shorebirds is closely linked to the snow melt in spring. We used two indices, the timing of snow melt and the spring 'green-up' to quantify inter annual variation and across year trends for two regions (North, South) of the bar-tailed godwit breeding range in Alaska.

We investigated the entire study period (2008–2020) as well as a shorter period (2008–2014) in which we tracked individuals with geolocators (see main text).

The Datasets

Timing of snowmelt: Remotely sensed IMS Daily Northern Hemisphere Snow and Ice Analysis data for the period 2008–2020 on a scale of 4 x 4 km were downloaded from the National Snow & Ice Data Center [1].

Spring "green-up": Remotely sensed NDVI data for the period 2008–2020 on a scale of 4 x 4 km were downloaded from NOAA STAR Center for Satellite Applications and Research [2]. The used noise reduced NDVI dataset is part of the blended-VHP products that is a re-processed Vegetation Health data set derived from VIIRS (2013–present) and AVHRR (1981–2012) GAC data. It was processed by the newly developed operational VHP system. The new VHP system was improved from GVI-x VH system and some changes/improvement were made to meet the requirement of operation and improve data quality. It can process GAC data from NOAA-19, as well as FRAC data from METOP A and METOP-B. It also produce vegetation health products from VIIRS on NPP and JPSS satellites. VHP system is operationaly running at NOAA Office of Satellite and Product Operations(OSPO) and providing official VHP products. This web site provides recent VH data as a backup/alternative data source. VHP product posted on this VH web site should be consistent to that released by OSPO.

Data analysis

Annotated R code and further explanations on environmental data manipulation and analyses are accessible via Zenodo, a public repository [3].

References

[1] IMS Daily Northern Hemisphere Snow and Ice Analysis at 1 km, 4 km, and 24 km Resolutions, Version 1. https://doi.org/10.7265/N52R3PMC

[2] https://www.star.nesdis.noaa.gov/smcd/emb/vci/VH/vh_ftp.php

[3] Jesse R. Conklin, Simeon Lisovski, & Phil F. Battley. (2021). Code from: Advancement in long-distance bird migration through individual plasticity in departure. Nature Communications. Zenodo. http://doi.org/10.5281/zenodo.5025715
