# Supplementary material for: Advancement in long-distance bird migration through individual plasticity in departure
Source: Nat Commun. 2021 Aug 6;12:4780. doi: 10.1038/s41467-021-25022-7 (PMC8346503; doi:10.1038/s41467-021-25022-7)
Supplement: Supplementary file 3 — Reporting Summary [file 41467_2021_25022_MOESM3_ESM.pdf]

nature research

Corresponding author(s): Jesse R. Conklin

Last updated by author(s): Jun 23, 2021

Reporting Summary

Nature Research wishes to improve the reproducibility of the work that we publish. This form provides structure for consistency and transparency in reporting. For further information on Nature Research policies, see Authors & Referees and the Editorial Policy Checklist .

Statistics

For all statistical analyses, confirm that the following items are present in the figure legend, table legend, main text, or Methods section.

n/a	Confirmed	
☐	☒	The exact sample size (*n*) for each experimental group/condition, given as a discrete number and unit of measurement
☐	☒	A statement on whether measurements were taken from distinct samples or whether the same sample was measured repeatedly
☐	☒	The statistical test(s) used AND whether they are one- or two-sided<br>*Only common tests should be described solely by name; describe more complex techniques in the Methods section.*
☐	☒	A description of all covariates tested
☐	☒	A description of any assumptions or corrections, such as tests of normality and adjustment for multiple comparisons
☐	☒	A full description of the statistical parameters including central tendency (e.g. means) or other basic estimates (e.g. regression coefficient) AND variation (e.g. standard deviation) or associated estimates of uncertainty (e.g. confidence intervals)
☐	☒	For null hypothesis testing, the test statistic (e.g. *F*, *t*, *r*) with confidence intervals, effect sizes, degrees of freedom and *P* value noted<br>*Give P values as exact values whenever suitable.*
☒	☐	For Bayesian analysis, information on the choice of priors and Markov chain Monte Carlo settings
☒	☐	For hierarchical and complex designs, identification of the appropriate level for tests and full reporting of outcomes
☒	☐	Estimates of effect sizes (e.g. Cohen's *d*, Pearson's *r*), indicating how they were calculated

Our web collection on statistics for biologists contains articles on many of the points above.

Software and code

Policy information about availability of computer code

Data collection	Geolocator data were acquired and processed using the open-access program BASTrak, and the R packages PolarGeolocation v.0.1.0 and SGAT v.0.1.3. Environmental data were accessed and manipulated using custom code.
Data analysis	Data were analyzed using the R packages lme4 v.1.1.21., arm v.1.11-2, and bbmle v.1.0.23.1.<br><br>All code for environmental analyses used in the paper have been deposited in Zenodo:<br>Jesse R. Conklin, Simeon Lisovski, & Phil F. Battley. (2021). Code from: Advancement in long-distance bird migration through individual plasticity in departure. Nature Communications. Zenodo. http://doi.org/10.5281/zenodo.5025715

For manuscripts utilizing custom algorithms or software that are central to the research but not yet described in published literature, software must be made available to editors/reviewers. We strongly encourage code deposition in a community repository (e.g. GitHub). See the Nature Research guidelines for submitting code & software for further information.

Data

Policy information about availability of data

All manuscripts must include a data availability statement. This statement should provide the following information, where applicable:
- Accession codes, unique identifiers, or web links for publicly available datasets
- A list of figures that have associated raw data
- A description of any restrictions on data availability

Minimum observational and geolocator-derived datasets used in the paper have been deposited in Zenodo:
Jesse R. Conklin, & Phil F. Battley. (2021). Data from: Advancement in long-distance bird migration through individual plasticity in departure (Version 0.0.1) [Data set]. Nature Communications. Zenodo. http://doi.org/10.5281/zenodo.5016733

Environmental datasets used in the paper are available online:

NSIDC. IMS Daily Northern Hemisphere Snow and Ice Analysis at 1 km, 4 km, and 24 km Resolutions, Version 1. https://doi.org/10.7265/N52R3PMC (2019).
NOAA. NOAA Center for Satellite Applications and Research. ftp://ftp.star.nesdis.noaa.gov/pub/corp/scsb/wguo/ (2018).

Field-specific reporting

Please select the one below that is the best fit for your research. If you are not sure, read the appropriate sections before making your selection.

☐ Life sciences ☐ Behavioural & social sciences ☒ Ecological, evolutionary & environmental sciences

For a reference copy of the document with all sections, see nature.com/documents/nr-reporting-summary-flat.pdf

Ecological, evolutionary & environmental sciences study design

All studies must disclose on these points even when the disclosure is negative.

Study description	For observational data, we tested for slope of migration date across 13 years, distinguishing within- and between-individual slopes using a mixed model approach (within-subject centering). For geolocator data, we tested for slope of date across years using linear mixed models with individual as a random factor and breeding region as a fixed factor. We described trends in environmental factors across years using linear regression.
Research sample	The study involves a sample of wild-living Bar-tailed Godwits (Limosa lapponica) at a single non-breeding site in New Zealand, representing the subspecies L. l. baueri that breeds in Alaska and winters in New Zealand and eastern Australia.
Sampling strategy	For population-level data, we did not sample, but observed or inferred all migratory departures that occurred at the study site. For individual-level data, we maintained a sufficient proportion (~35%) of the local population marked with field-readable band combinations to represent all potential migration strategies in the population (by sex, size, and breeding region in Alaska), while minimizing capture-related disturbance and ensuring that daily observations were practical for a single observer to perform (44-81 individuals per year). For light-level geolocator data, sample sizes were limited by available budget (i.e, cost of units and deployment) and practical concerns (i.e., how many instrumented birds returned after migration, and how many we could re-capture without undue disturbance).
Data collection	All observational data were collected by the lead author, using a combination of a 20–60x spotting scope and digital camera w/400 mm lens.
Timing and spatial scale	Godwits were observed daily throughout the potential migratory departure periods (late Feb-early Apr of the years 2008-2020) until all individual birds departed on migration. Observations were semi-continuous (depending on bird behavior) in all conditions during daylight hours. Geolocators were deployed in the non-breeding seasons (October-March) of 2008, 2009, 2013, and 2014 and retrieved in subsequent non-breeding seasons, therefore providing northward migration data for those 4 years.
Data exclusions	No data were excluded.
Reproducibility	No repetition of such a study is feasible.
Randomization	There were no assigned treatments in the study.
Blinding	Blinding is inapplicable to the study, as there were no assigned treatments and all data are based on objectively observed outcomes.

Did the study involve field work? ☒ Yes ☐ No

Field work, collection and transport

Field conditions	Fieldwork involved direct observation of wild birds from the perimeter or exposed mudflat of a small intertidal estuary, from late February until early April, during daylight hours in all weather conditions.
Location	The Manawatu River estuary (40.47°S, 175.22°E), North Island, New Zealand
Access and import/export	The site is a public area with no restriction to access. There were no samples collected in the study.
Disturbance	Observational fieldwork was conducted at a distance taking into account bird behavior, and thus generally created no disturbance. Cannon-net captures were conducted on only 1-2 days per 6-month non-breeding season, minimizing disturbance to the local population. Captures were conducted at high tide, when birds were not foraging, to reduce potential impacts.

Reporting for specific materials, systems and methods

We require information from authors about some types of materials, experimental systems and methods used in many studies. Here, indicate whether each material, system or method listed is relevant to your study. If you are not sure if a list item applies to your research, read the appropriate section before selecting a response.

Materials & experimental systems

n/a	Involved in the study
☒	☐ Antibodies
☒	☐ Eukaryotic cell lines
☒	☐ Palaeontology
☐	☒ Animals and other organisms
☒	☐ Human research participants
☒	☐ Clinical data

Methods

n/a	Involved in the study
☒	☐ ChIP-seq
☒	☐ Flow cytometry
☒	☐ MRI-based neuroimaging

Animals and other organisms

Policy information about studies involving animals; ARRIVE guidelines recommended for reporting animal research

Laboratory animals	No laboratory animals were involved in the study.
Wild animals	Bar-tailed Godwits (Limosa lapponica) were captured by cannon-net at a high-tide roost. Birds were measured and banded with field-observable leg colorbands and released at the site within 2 hours of capture. A subset of individuals was equipped with tibia-mounted geolocators and recaptured 1-2 years later to retrieve the data.
Field-collected samples	Study did not involve samples collected from the field.
Ethics oversight	Fieldwork was conducted with Massey University Animal Ethics Committee approval (#07/163, 12/90 and 16/117) and appropriate New Zealand Department of Conservation permits (Banding permit 2007/39, 35503-FAU, 38111-FAU).

Note that full information on the approval of the study protocol must also be provided in the manuscript.
